# Effectiveness of problem-based learning in Chinese pharmacy education: a meta-analysis

**DOI:** 10.1186/s12909-016-0546-z

**Published:** 2016-01-19

**Authors:** Jiyin Zhou, Shiwen Zhou, Chunji Huang, Rufu Xu, Zuo Zhang, Shengya Zeng, Guisheng Qian

**Affiliations:** National Drug Clinical Trial Institution, Xinqiao Hospital, Third Military Medical University, Chongqing, 400037 People’s Republic of China; Institute of Respiratory Diseases, Xinqiao Hospital, Third Military Medical University, Chongqing, 400037 People’s Republic of China

**Keywords:** Pharmacy education, Problem-based learning, China

## Abstract

**Background:**

This review provides a critical overview of problem-based learning (PBL) practices in Chinese pharmacy education. PBL has yet to be widely applied in pharmaceutical education in China. The results of those studies that have been conducted are published in Chinese and thus may not be easily accessible to international researchers. Therefore, this meta-analysis was carried out to review the effectiveness of PBL.

**Methods:**

Databases were searched for studies in accordance with the inclusion criteria. Two reviewers independently performed the study identification and data extraction. A meta-analysis was conducted using Revman 5.3 software.

**Results:**

Sixteen randomized controlled trials were included. The meta-analysis revealed that PBL had a positive association with higher theoretical scores (SMD = 1.17, 95 % CI [0.77, 11.57], *P* < 0.00001). The questionnaire results show that PBL methods are superior to conventional teaching methods in improving students’ learning interest, independent analysis skills, scope of knowledge, self-study, team spirit, and oral expression.

**Conclusions:**

This meta-analysis indicates that PBL pedagogy is superior to traditional lecture-based teaching in Chinese pharmacy education. PBL methods could be an optional, supplementary method of pharmaceutical teaching in China. However, Chinese pharmacy colleges and universities should revise PBL curricula according to their own needs, which would maximize the effectiveness of PBL.

**Electronic supplementary material:**

The online version of this article (doi:10.1186/s12909-016-0546-z) contains supplementary material, which is available to authorized users.

## Background

Problem-based learning (PBL) is an educational innovation developed and first implemented in the 1960s in response to dissatisfaction with conventional medical education practices [1–5]. Many researchers have investigated the effectiveness of PBL in medical school curricula [6, 7] and findings indicate that PBL can contribute to knowledge retention, student satisfaction, motivation, and critical thinking [8, 9]. The first published application of PBL in pharmacy was a patient-oriented problem-solving instruction module for pharmacy students developed in 1983 by Love and Shumway [10]. Since then, PBL has been applied in pharmaceutical education courses and several studies have reported results from the use of this educational method [4, 11, 12].

However, PBL is not often used in Chinese pharmacy education. There are several reasons for this. First, pharmacy tuition fees are less than 10,000 RMB (around 1600 USD). The Chinese government provides all the equipment and materials that pharmacy students require. The application of PBL is restricted by a shortage of educational equipment and materials. Second, both the standards and levels of pharmacy education vary significantly among different institutions in China. Third, almost all education is characterized by traditional educational methods that have been used for decades. Traditional education is teacher-oriented and places too much emphasis on disseminating factual knowledge and on passive student learning. Fourth, Chinese curricula are arranged differently from those of other countries. After completing all the pharmacy courses in 3 to 3.5 years, pharmacy students begin practical skills training. As teaching courses are limited, pharmacy colleges and universities tend to employ conventional teaching methods rather than innovative methods such as PBL. Furthermore, humility and courtesy are important qualities of Chinese communication. Research based on the Myers-Briggs Type Indicator (which measures differences in individuals’ psychological preferences for particular cognitive functions, such as perception and judgment [13]) has shown that Chinese pharmacy students with stronger introversion gain higher grades than those scoring strongly on extraversion [14]. Because Chinese people are taught to be humble and to comply with their elders, they are more likely to express an introspective, quiet, and conservative personality [15]. Studies using the Myers-Briggs learning styles have shown that pharmacy service professionals in Western countries have an obvious preference for the judging style [16, 17]. Introverted individuals participate less in open discussions because they are reluctant to express their opinions, especially if their opinions differ from those of others.

Because of the reasons discussed, China has been slower to incorporate PBL into pharmacy education than other countries, such as the United States. As there is great demand for highly qualified pharmacists in China, curriculum reform that includes the application of PBL teaching methods is urgently needed. In recent years, many Chinese pharmacy institutions have made tentative steps in PBL pedagogy. Studies on the effectiveness of PBL methods in pharmacy education in China have reported positive outcomes [18–33]. One recent systematic review and meta-analysis shows that the PBL method improved pharmacy students’ knowledge in the United States, the United Kingdom, and Canada [3]. However, the benefits of PBL for undergraduate pharmacy students in China have not been clearly disseminated. Papers on PBL in Chinese pharmacy education have not been published in English and therefore cannot be accessed by non-Chinese-speaking researchers. The aim of this meta-analysis was to review the effectiveness of PBL in Chinese pharmacy education and to disseminate this research more widely to international education researchers. To evaluate the overall effectiveness of studies of PBL pedagogy, we used only studies conducted in China and focused on student-centered pharmacy education programs rather than traditional teaching methods.

## Methods

### Study design

We conducted a systematic review and meta-analysis following the guidelines of the Cochrane Handbook for Systematic Reviews of Interventions [34] and the Preferred Reporting Items for Systematic Review and Meta-Analysis Protocols (PRISMA-P) 2015 statement recommendations (Additional file 1) [35].

### Search strategy

We searched the electronic databases Chinese National Knowledge Infrastructure (CNKI), VIP Information (Chinese database), Wanfang Data (Chinese database), and Chinese Biomedical Literature (CBM). English-language computerized databases, such as PubMed, EMBASE, and the Cochrane Database, were also searched. Next, the reference list of selected articles was reviewed for additional related reports. The search was restricted to the period 1965 to December 2014, as PBL originated at the McMaster School of Medicine in Canada in 1965 [36]. The mesh-terms or key words (“problem-based learning”) AND (“pharmac*” OR “pharmac* education” OR “pharmac* students”) were used for the search string. The literature search was conducted in April 2014; we updated the search on November 29, 2014. A total of 419 abstracts were retrieved.

### Study selection

Two reviewers independently selected the studies; any discrepancies were resolved by discussion. The studies were first selected according to the title and abstract. We included articles if they (1) investigated students of pharmacy institutions in China; (2) described randomized controlled trials; (3) included pharmacy students from nonpharmacy institutions; (4) used PBL as an educational approach in the intervention group; (5) used traditional lectures in the control group and exposed neither group to supplementary teaching methods that could have an impact on the results; (6) evaluated theoretical scores and questionnaires as outcomes; and (7) reported the sample size and the mean difference in theoretical scores for the intervention group and control group. Articles were excluded if they (1) were non-randomized controlled trials; (2) included subjects other than pharmacy students; (3) utilized interventions other than PBL; and (4) had incomplete data, such as not reporting the mean difference in theoretical scores. We finally selected 13 randomized controlled trials.

### Data extraction

Data were extracted by two independent reviewers. Disagreements about eligibility were resolved by consensus. For each study, the following information was extracted in the current analysis: the first author, publication year, the pharmacy disciplines involved, sample size (intervention group and control group), characteristics of participants, intervention method, teaching method for the control group, outcomes (the mean difference in theoretical examination scores for the intervention group and control group), time of outcome measure, outcomes measured, and length of intervention.

### Quality assessment

The quality of the included studies was assessed as adequate, uncertain, or inadequate by two reviewers and was based on the six general sources of bias described in the Cochrane Handbook for Systematic Reviews of Interventions [34]. The following quality items were checked: adequacy of the generation of the allocation sequence, concealment of allocation, blinding procedures, incomplete outcome data, selective outcome reporting, and other sources of bias. Information addressed by these items was obtained from the published reports and authors were contacted if additional information was required [3]. The Cochrane Collaboration’s tool for assessing risk of bias is available on line at http://handbook.cochrane.org/ [34].

### Statistical analysis

Review Manager 5.3 software [37] was used to test the data for heterogeneity and to carry out the meta-analysis. We analyzed the theoretical scores of the PBL groups and the control groups. As continuous data from different scales were extracted, the standardized mean difference (SMD) was calculated for effect size based on sample size [38] and 95 % confidence intervals for each study, and for the pooled studies using variance analysis. Weighted mean differences and 95 % confidence intervals were calculated for continuous data from the same scale. A two-sided *P* value of less than 0.05 was regarded as significant for all analyses. We used two meta-analysis models. A fixed-effects model was used to pool data if there was no heterogeneity, otherwise we used a random-effects model. Heterogeneity was considered significant for a *P* value of Cochran’s Q statistic <0.10 and I^2^ > 50 % [39, 40]. I^2^ is the percentage of variation attributed to heterogeneity and is easily interpreted. An I^2^ statistic of 25–50 % was considered low, 50–75 % was considered moderate, and ≥75 % was considered high.

If heterogeneity was revealed, we conducted a sensitivity analysis to assess if this significantly altered the results of the meta-analysis. We performed the sensitivity analysis by excluding studies associated with heterogeneity and then recalculating the pooled estimates for the remaining studies. However, this did not significantly alter the results.

Publication bias was assessed with a funnel plot and Egger’s test of asymmetry [41] using STATA 13.0. The funnel plot shapes did not reveal any obvious evidence of asymmetry, and all *P* values for Egger’s tests were more than 0.05, providing statistical evidence of the funnel plot symmetry.

## Results

### The search results

**Fig. 1:**
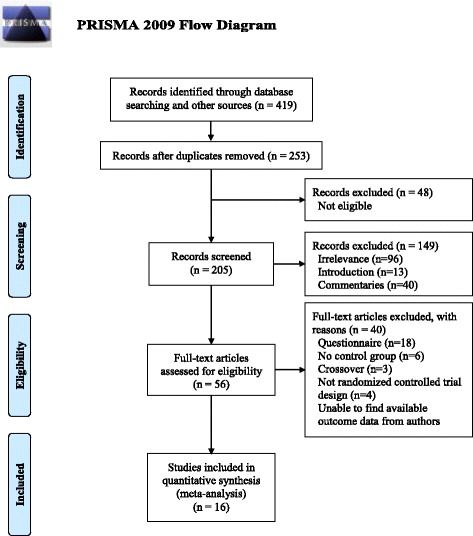
Inclusion and exclusion of randomized controlled trials of PBL in pharmacy education in mainland China

The literature search identified 419 abstracts, out of which 166 duplicates were removed. A further 48 articles were excluded after reading the titles and abstracts, among which 40 were review articles and 96 were not relevant to this investigation. In accordance with the inclusion and exclusion criteria, relevant full-text articles (*n* = 56) were assessed for eligibility. Eighteen studies did not measure theoretical scores and only used questionnaires, six did not include a control group, three used PBL and other teaching methods for the intervention group, four were non-randomized controlled trials, and nine did not report detailed outcome data. We e-mailed the corresponding authors to ask for the available outcome data but received no reply. Thus, 16 articles were included in the meta-analysis. The data abstraction process is shown in Fig. 1.

### Characteristics of included studies

Sixteen articles [18–33] representing 17 studies were included in the meta-analysis. They were all published between 2006 and 2014 and were in Chinese. The sample sizes ranged from 28 to 112 participants and the pooled sample size was 1826 (PBL group = 929, control group = 897). The studies examined teaching methods in different areas of pharmacy, as follows: three studies of pharmaceutical analysis [20, 24, 28]; three of natural medicine chemistry [18, 30, 33]; two of biopharmaceutics and pharmacokinetics [22, 31]; one of pharmaceutical formulation design [19]; one of pharmaceutical affairs, law, and regulation [21]; one of Chinese materia medica pharmacology [23]; one of basic pharmacy [25]; one of pharmaceutical botany [27]; one of pharmaceutical literature retrieval [26]; one of organic chemistry [29]; and one of pharmaceutical molecular biology [32]. All study outcomes were measured using theoretical scores and questionnaire surveys after PBL teaching. The length of intervention varied from one semester to two semesters. Table 1 shows the characteristics of the 16 included studies.

**Table 1:** The characteristics of the 16 included studies

Study ID	Pharmacy disciplines	Sample size (PBL/LBL)	Participant characteristics	Interventions	Comparator teaching approach	Duration of intervention
Che [18]	Natural products chemistry	40/40	Year three pharmacy students from two classes at a college	Educational approach: PBL.	Lecturing	One semester
				Total of 12 class hours.		
				The teaching process included preview, search information, laying out the problem, answers, discussion, and effectiveness evaluation.		
Du et al. [19]	Pharmaceutical formulation design	43/48	Year four pharmacy students from one class at a college (43 females and 48 males)	Educational approach: PBL.	Lecturing	One semester
				There were two PBL tutorial groups and each group consisted of either 21 or 22 students.		
				The teaching process included preview, search information, laying out the problem, answers, discussion, and effectiveness evaluation.		
Fang et al. [20]	Pharmaceutical analysis	90/95	Seventh semester pharmacy students at a university	Educational approach: PBL.	Lecturing	One semester
				There were nine PBL tutorial groups and each group consisted of 10 students.		
				The teaching process included laying out the learning goal, content and requirements, self-study, search information, group discussion, answers, conclusion, and effectiveness evaluation.		
Ge et al. [21]	Pharmaceutical affairs, law, and regulation	57/37	Year four pharmacy students at a university	Educational approach: PBL.	Lecturing. Total of 24 class hours.	One semester
				Total of 24 class hours.		
				The teaching process included laying out the problem, self-study, discussion (10–20 min), and teacher summary (15 min).		
Huang et al. [22]	Biopharmaceutics and pharmacokinetics	112/91	Year three pharmacy students from two classes at a college	Educational approach: PBL.	Lecturing. Each group consisted of 16 students.	One semester
				Each group consisted of two students.		
				The teaching process included designing cases, group discussion, calculating an experimental program, doing experiments, score grading, and conclusion.		
Li [23]	Pharmacology of Chinese materia medica	54/54	Fifth semester pharmacy students from four classes at a university	Educational approach: PBL.	Lecturing	One semester
				Each group consisted of either six or seven students.		
				The teaching process included designing cases, group discussion, calculating an experimental program, doing experiments, score grading, and conclusion.		
Pu [24]	Pharmaceutical analysis	49/46	Fifth semester pharmacy students from two classes at a higher vocational college	Educational approach: PBL.	Lecturing	One semester
				There were seven PBL tutorial groups and each group consisted of seven students.		
				The teaching process included laying out the problem, search information, group discussion, summary, and effectiveness evaluation.		
Shen [25]	Basic pharmacy	60/60	Year one pharmacy students at a college. Age: 17–21 years, mean age 24.7 years (SD, 2.3); 57 females and 63 males	Educational approach: PBL.	Lecturing	One semester
				There were six PBL tutorial groups and each group consisted of 10 students.		
				The teaching process included laying out an open problem, self-study, group discussion, finding solutions, and effectiveness evaluation.		
Wang et al. [27]	Pharmaceutical botany	43/43	Year one pharmacy students from one class at a university	Educational approach: PBL.	Lecturing	One semester
				The teaching process included laying out the problem, group discussion, answering, and scoring.		
Wang et al. [26]	Pharmaceutical literature retrieval	50/51	Year three pharmacy students from two classes at a university; 55 females and 46 males	Educational approach: PBL.	Lecturing. Total of 32 class hours.	Two semesters
				Each group consisted of 6–10 students.		
				Total of 32 class hours.		
				The teaching process included subject design, laying out the problem, search information, discussion, retrospection, summary, and comments.		
Yang et al. [28]	Pharmaceutical analysis	30/28	Year three pharmacy students from two classes at a vocational college	Educational approach: PBL.	Lecturing	One semester
				Each group consisted of either seven or eight students.		
				The teaching process included laying out the problem, self-study, experimental design, conducting an experiment, discussion, analyzing results, summary, and evaluation.		
Yang & Li [29]	Organic chemistry	102/102	Year two pharmacy students at a college	Educational approach: PBL.	Lecturing	One semester
				Each group consisted of 6–8 students.		
				The teaching process included laying out the problem, reading guidance, self-study, group discussion, and summary.		
Yu et al. [30]	Natural medicine chemistry	40/40	Year three pharmacy students from one class at a university	Educational approach: PBL.	Lecturing	One semester
				There were eight PBL groups and each group consisted of five students.		
				The teaching process included information searching, group discussion, designing an experiment, conducting an experiment, evaluating, and completing the experimental program.		
Zhang [31]	Biopharmaceutics and pharmacokinetics	44/45	Year three pharmacy students from two classes at a college	Educational approach: PBL.	Lecturing	One semester
				Each group consisted of either four or five students.		
				The teaching process included laying out the problem, information searching, group discussion, and summary.		
Zhang et al. [32]	Pharmaceutical molecular biology	30/30	Year two pharmacy students at a college	Educational approach: PBL.	Lecturing. There were 40 theoretical class hours and 18 experimental class hours (40 min per lecture).	One semester
				There were 40 theoretical class hours and 18 experimental class hours (40 min per lecture).		
				The teaching process included laying out the problem, self-study, discussion, and answering questions.		
Zhuo & Wu [33]	Natural products chemistry	85/87	Year three pharmacy students at a college	Educational approach: PBL.	Lecturing. There were 9 theoretical class hours.	One semester
				There were 9 theoretical class hours.		
				The teaching process included laying out the problem, information searching, group discussion, and summary.		

*PBL* problem-based learning, *LBL* lecture-based learning

### Study quality

Table 2 shows the risk of bias assessment of the 16 included studies. Most studies had a low risk of bias across the six domains. The allocation sequence in one study [24] was adequately generated by random numbers. In another study [19], the allocation sequence was generated by odd and even numbers based on the college entrance examination scores. The allocation sequence of four studies [21, 23, 28, 31] was based on the preference of the researchers, who decided which students to assign to the experimental or control groups. The other 10 studies did not report the allocation method used, so this information was not available. So it was not possible to assess the blinding of the students and tutors to the interventions. All studies reported complete outcome data. We assessed whether each study was free from selective outcome reporting by checking whether all outcomes mentioned were adequately reported in the results section. All studies adequately reported the results. All studies seemed free from “other sources of bias” as defined in the Cochrane Collaboration’s domain-based evaluation.

**Table 2:** Risk of bias assessment of the 16 included randomized controlled studies

Study ID	Randomization	Allocation concealment	Blinding	Incomplete data report	Selective data report	Other bias
			(a) participants blinded			
			(b) operator blinded			
			(c) assessor blinded			
			(d) statistician blinded			
Che [18]	Yes	Unclear	(a) Unclear	None	None	None
			(b) Unclear			
			(c) Unclear			
			(d) Unclear			
Du et al. [19]	Yes	Unclear	(a) Unclear	None	None	None
			(b) Unclear			
			(c) Unclear			
			(d) Unclear			
Fang et al. [20]	Yes	Unclear	(a) Unclear	None	None	None
			(b) Unclear			
			(c) Unclear			
			(d) Unclear			
Ge et al. [21]	Unclear	Unclear	(a) Unclear	None	None	None
			(b) Unclear			
			(c) Unclear			
			(d) Unclear			
Huang et al. [22]	Unclear	Unclear	(a) Unclear	None	None	None
			(b) Unclear			
			(c) Unclear			
			(d) Unclear			
Li [23]	Unclear	Unclear	(a) Unclear	None	None	None
			(b) Unclear			
			(c) Unclear			
			(d) Unclear			
Pu [24]	Yes	Unclear	(a) Unclear	None	None	None
			(b) Unclear			
			(c) Unclear			
			(d) Unclear			
Shen [25]	Yes	Unclear	(a) Unclear	None	None	None
			(b) Unclear			
			(c) Unclear			
			(d) Unclear			
Wang et al. [27]	Yes	Unclear	(a) Unclear	None	None	None
			(b) Unclear			
			(c) Unclear			
			(d) Unclear			
Wang et al. [26]	Yes	Unclear	(a) Unclear	None	None	None
			(b) Unclear			
			(c) Unclear			
			(d) Unclear			
Yang et al. [28]	Yes	Unclear	(a) Unclear	None	None	None
			(b) Unclear			
			(c) Unclear			
			(d) Unclear			
Yang & Li [29]	Unclear	Unclear	(a) Unclear	None	None	None
			(b) Unclear			
			(c) Unclear			
			(d) Unclear			
Yu et al. [30]	Yes	Unclear	(a) Unclear	None	None	None
			(b) Unclear			
			(c) Unclear			
			(d) Unclear			
Zhang [31]	Yes	Unclear	(a) Unclear	None	None	None
			(b) Unclear			
			(c) Unclear			
			(d) Unclear			
Zhang et al. [32]	Unclear	Unclear	(a) Unclear	None	None	None
			(b) Unclear			
			(c) Unclear			
			(d) Unclear			
Zhuo & Wu [33]	Yes	Unclear	(a) Unclear	None	None	None
			(b) Unclear			
			(c) Unclear			
			(d) Unclear			

### Effects of interventions on theoretical examination scores

**Fig. 2:**
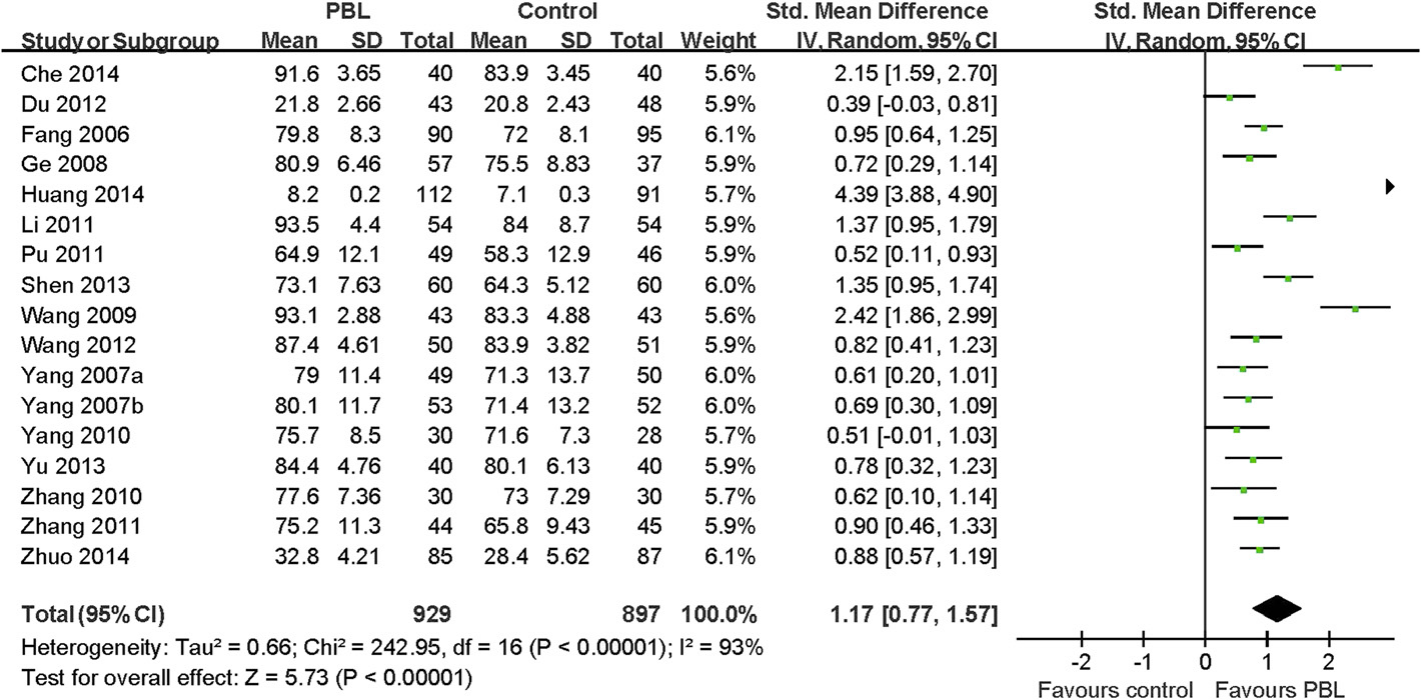
Meta-analysis and forest plot of theoretical scores for PBL compared with traditional lectures

The effects of the PBL methods were evaluated using both quantitative data and description. Sixteen studies involving 1826 participants (PBL group = 929, control group = 897) reported theoretical scores. Yang and Li [29] reported two types of outcome data; thus, there were 17 reports of outcome data in the meta-analysis. This meta-analysis reviews the effects of PBL on theoretical scores, which are more objective than other indices. All studies showed statistically significant differences between the PBL and the control groups in pharmacy students’ theoretical scores. There was high heterogeneity (I^2^ = 93 %, *P <* 0.00001); thus, the random-effects model was used. The pooled effect size showed a significant difference in theoretical scores (SMD = 1.17, 95 % CI [0.77, 1.57], *P <* 0.00001) in favor of PBL, compared with traditional lectures (Fig. 2). The results indicate that PBL improves students’ academic achievement. The fixed-effects model was also applied to pool the data. The pooled effects favored the PBL group (SMD = 1.06, 95 % CI [0.95, 1.16], *P <* 0.00001).

Because we observed heterogeneity between studies reporting theoretical scores, a sensitivity analysis was carried out to verify the reliability of the results. This was performed using sequential omission of individual studies. After excluding five studies [21–23, 28, 31] with inadequate generation of a randomized sequence from the analyses of theoretical scores, the pooled effect size favored the PBL group (SMD = 1.06, 95 % CI [0.77, 1.35], *P* < 0.00001) and did not change the effects observed in the primary analysis.

**Fig. 3:**
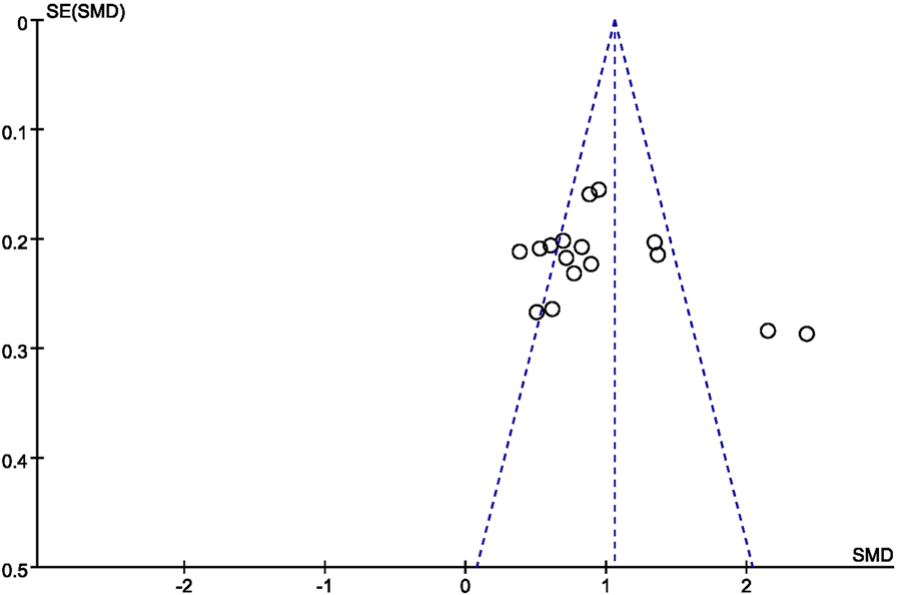
Funnel plot analysis of theoretical scores

The funnel plot for the 17 reports on the theoretical scores analysis is shown in Fig. 3. The shape of the funnel plot is symmetrical indicating no significant publication bias.

### Effects of interventions assessed by questionnaires

According to the questionnaire results, most students at the PBL pilot institutions showed more enthusiasm for PBL methods than for conventional teaching methods. The questionnaire results also show that the PBL methods are superior to the conventional teaching methods in improving students’ learning interest, independent analysis skills, scope of knowledge, self-study, team spirit, and oral expression.

## Discussion

The pharmacy students in the PBL groups had better theoretical examination scores than those in the traditional learning method groups. This result is in accordance with the findings of a previous review of the efficiency of PBL learning methods in pharmacy education in United States, United Kingdom, and Canada, but not in China [3]. To our knowledge, this is the first systematic review and meta-analysis of the effects of PBL in Chinese pharmaceutical education.

There are several possible reasons why pharmacy students who received PBL teaching had higher theoretical examination scores that those who received traditional teaching. First, the main difference between PBL and a traditional curriculum is that teachers and other faculty personnel in a PBL program engage more with students [42–44]. Second, the PBL teaching model can inspire students to engage in proactive learning and thinking initiatives, facilitating a stronger grasp of experimental processes and logic. Third, PBL encourages students to think about and resolve practical problems. It may also help pharmacy students to apply their knowledge to work situations, improve their professional reasoning, and encourage self-directed learning during their professional careers [45, 46].

The questionnaire results indicated that students showed more enthusiasm for PBL than for traditional learning. The questionnaire results also show that PBL methods are superior to conventional teaching methods in improving students’ learning interest, independent analysis skills, scope of knowledge, self-study, team spirit, and oral expression. In PBL teaching methods, pharmacy students were asked to laying out the problem, search information, group discussion, summary, and effectiveness evaluation, which are the basic progress for PBL teaching methods but not for conventional teaching methods. After the related training process of PBL teaching methods, the pharmacy students showed more enthusiasm and raised abilities of learning interest, self-analysis, self-study, team collaboration, and spoken expression.

Learning is most effective when students are actively involved in PBL [47]. The present results for pharmacy education are consistent with recent research in nursing education [48], medical education [8], and Chinese dental education [49]. This suggests that PBL is the best education method for healthcare courses [50]. The main obstacles to implementing PBL include training of teachers and other staff and necessary decreases in class size, which could raise pharmacy education fees [45, 51]. Because most of the 16 studies reviewed here used random allocation or blinding methods, the methodological quality of the included articles was relatively high. Differences of age, gender, and scores on college entrance exams between the PBL and control groups were mentioned in most studies, so the sampling error was minimal. Those studies that did not describe random allocation or blinding methods were not included in the analysis.

There was an obvious heterogeneity among the 16 pooled studies for the theoretical scores, which may have resulted from the following factors. First, there were differences in the educational levels of the students and the pharmacy schools in the included studies. In the Chinese pharmacy education system, pharmacy schools are established by various colleges or universities, which provide different levels of education. The criteria for admission of pharmacy students are not based on a standard examination, such as the Pharmacy College Admission Test used in the United States. For example, of the 16 pooled studies, 10 studies [18, 19, 22, 24, 25, 28, 29, 31–33] were conducted in colleges and the other six studies [20, 21, 23, 26, 27, 30] were conducted in universities (higher level institutions). This diversity is a fundamental cause of the heterogeneity among those studies. Second, the pooled studies focus on different pharmacy disciplines. In this review, we synthesized these studies to assess the total effectiveness; however, these differences in discipline may have resulted in heterogeneity. The third reason concerns the examination method. Sixteen studies employed different examinations. There are no unified criteria for the evaluation of the effectiveness of PBL pedagogy on theoretical knowledge. Fourth, the pharmacy institutions did not use a standard PBL pedagogy; these different teaching methods and objectives may have resulted in different effects.

In China, the implementation of the PBL method is still at an early stage. Some pharmacy colleges and universities have made tentative steps to utilize PBL methods in pharmacy education. Because PBL is an effective way to prepare pharmacy students for careers in the pharmaceutical industry, it should be used more widely in China, rather than only being applied in an exploratory way. However, it is difficult for pharmacy colleges and universities in China to model PBL teaching methods that have been successful in other countries, because Chinese pharmacy education is characterized by special teaching conditions. With the exception of curricula for particular specialties, PBL should be aimed at educating general pharmacists to provide recommendations for the most optimized treatment schedules for patients.

### Limitations and future studies

The methodological quality of the included articles was low. The included studies involved research in the field of pharmacy education, so it was impossible for the researchers to implement allocation concealment and blinding. Although the studies we included were randomized controlled trials, and two investigators examined the studies and extracted the data independently, selection bias and performance bias were unavoidable. Moreover, there are no standard criteria for assessing the effectiveness of PBL pedagogy. Therefore, this meta-analysis is likely to contain measurement bias. However, we found no evidence of attrition bias. Lastly, the methodological qualities of studies in the field of pharmacy education are not comparable to those of pharmaceutical science research for multiple reasons. However, this review does provide information about implementing PBL pedagogy and suggests the utilization of a more standardized method to assess PBL.

The 16 included articles did not adopt uniform outcome measures. There was no standard examination to test the theoretical outcomes, which are designed to reflect precisely the effect of the PBL pedagogy. Before a standard evaluation system of PBL pedagogy is established, existing standard examinations, such as the North American Pharmacist Licensure Examination (NAPLEX) and the Multistate Pharmacy Jurisprudence Examination (MPJE) in the United States, or corresponding examinations in other countries, could be used as evaluation tests. In 1993, Vernon and Blake [52] conducted a meta-analysis of PBL in medical education using the National Board Medical Examination (NBME) as a theoretical test. The scaled scores and pass rate on the North American Pharmacist Licensure Examination for the University of Mississippi were both above the national scores in all reporting periods from 2001 to 2005 except one [53]. In China, a standard examination such as the Chinese Pharmacy License Examination could be adopted as the standard examination for PBL pedagogy evaluation. High-quality randomized trials are needed to eliminate bias. Most of the 16 included studies did not describe random allocation methods in detail. PBL curriculum design should include precise educational objectives of PBL pedagogy. The impact of PBL on postdoctoral plans and extracurricular activities, especially research, should also be assessed in future studies [54].

Another limitation of this review was the absence of studies that assessed effects on the professional achievements of pharmacists who experienced the PBL method compared with those who received traditional methods of instruction. The present findings indicate that PBL students demonstrated higher theoretical examination scores; however, we cannot infer that these students will become better professionals.

Future research should prioritize experimental designs that evaluate effects that are more directly related to professional effectiveness and workplace performance. Greater recording and publication of evidence about PBL implementation would facilitate the adoption of this method without substantially raising fees.

## Conclusions

The PBL curriculum seems to improve the academic performance of pharmacy students when compared with the traditional method of instruction. However, the heterogeneity of the selected studies needs to be considered. Most of the questionnaire surveys indicated the method’s positive effects on students’ learning interest, independent analysis skills, scope of knowledge, self-study, team spirit, and oral expression. Directors and teachers of pharmacy courses should consider gradually implementing PBL methods into their programs, because PBL pedagogy may be superior to traditional lecture-based teaching. PBL methods could be an optional, supplementary method for pharmacy teaching models in China. However, Chinese pharmacy schools should devise PBL curricula according to their own needs, which would optimize the effectiveness of PBL. Reporting the results from such initiatives is likely to improve the quality of the existing evidence in support of PBL methods.

## Additional file

Additional file 1:
**PRISMA 2009 Checklist.** (DOC 65 kb)

## Abbreviations

PBL: Problem-based learning
SMD: Standardized mean difference
